# Cochlear Implantation: The Volumetric Measurement of Vestibular Aqueduct and Gusher Prediction

**DOI:** 10.3390/jpm13020171

**Published:** 2023-01-19

**Authors:** Asma Alahmadi, Yassin Abdelsamad, Fida Almuhawas, Nezar Hamed, Marzouqi Salamah, Abdulrahman Alsanosi

**Affiliations:** 1 King Abdullah Ear Specialist Center (KAESC), College of Medicine, King Saud University Medical City (KSUMC), King Saud University, Riyadh 11411, Saudi Arabia; 2 Research Department, MED-EL GmbH, Riyadh 11411, Saudi Arabia

**Keywords:** cochlear implantation, enlarged vestibular aqueduct, Mondini dysplasia, cochlear metrics

## Abstract

This study aimed to validate the role of 3D segmentation in measuring the volume of the vestibular aqueduct (VAD), and the inner ear, and to study the correlation between VAD volume and VAD linear measurements at the midpoint and operculum. The correlation with other cochlear metrics was also studied. We retrospectively recruited 21 children (42 ears) diagnosed with Mondini dysplasia (MD) plus enlarged vestibular aqueduct (EVA) from 2009 to 2021 and who underwent cochlear implantation (CI). Patients’ sociodemographic data were collected, and linear cochlear metrics were measured using Otoplan. Vestibular aqueduct width and vestibular aqueduct and inner ear volumes were measured by two independent neuro-otologists using 3D segmentation software (version 4.11.20210226) and high-resolution CT. We also conducted a regression analysis to determine the association between these variables and CT VAD and inner ear volumes. Among the 33 cochlear implanted ears, 13 ears had a gusher (39.4%). Regarding CT inner ear volume, we found that gender, age, A-value, and VAD at the operculum were statistically significant (*p*-Value = 0.003, <0.001, 0.031, and 0.027, respectively) by regression analysis. Moreover, we found that Age, H value, VAD at the midpoint, and VAD at the operculum were significant predictors of CT VAD volume (*p*-Value < 0.04). Finally, gender (OR: 0.092; 95%CI: 0.009–0.982; *p*-Value = 0.048) and VAD at the midpoint (OR: 0.106; 95%CI: 0.015–0.735; *p*-Value = 0.023) were significant predictors of gusher risk. Patients’ gusher risk was significantly differentiated by gender and VAD width at the midpoint.

## 1. Introduction

Hearing impairment is considered one of the most common congenital anomalies worldwide, estimated to present in 2–4 out of every 1000 children [1,2]. Accordingly, neonatal screening programs have been thoroughly integrated into healthcare systems throughout the past [3]. Most congenital hearing deficits have a genetic etiology and are attributed to defects in the Connexin 26/GJB2 gene [4,5,6,7,8]. Enlarged vestibular aqueduct (EVA) is the most common inner ear anomaly, which causes 20% of sensorineural hearing loss (SNHL) [9,10,11]. It is usually linked to fluctuating and progressive SNHL starting in early childhood. Moreover, it is commonly related to abrupt onset or progression due to minor head trauma or sudden barometric pressure variations.

Moreover, EVA could be isolated or associated with other inner ear anomalies such as Mondini deformity (MD) or Incomplete partition type II [12]. MD develops due to an embryogenic anomaly happening in the seventh week [13,14] and it can be associated with different genetic mutations, most commonly mutation in the PDS gene [15,16,17]. Multichannel cochlear implantation (CI) in MD patients was first performed by Silverstein and colleagues [18]. This procedure helped restore hearing to close-to-normal levels in multiple MD cohorts worldwide [19,20,21,22].

Accordingly, it is crucial to establish a timely and accurate diagnosis of the affected patients to enhance their communication and clinical outcomes. This is even more vital, especially in cases with rapid function loss following barotrauma or traumatic head injuries [4,5,6,7]. In 1978, Valvassori and Clemis suggested the original criterion for EVA syndrome (EVAS) diagnosis to be a VA width greater than 1.5 mm at the midpoint [4,5,6,7,8]. Since then, various criteria have been described, ranging between 2 mm at the midpoint and 4 mm at the operculum [23]. Moreover, the preferred method to diagnose EVA has been recently reported to be the Cincinnati criteria. Evidence from previous studies validated these criteria to refine EVA size (being ≥1 mm or 2 mm at the midpoint or the operculum, respectively), based on internal auditory canal CT measurements [16,24]. In addition, the Cincinnati criteria are more sensitive in diagnosing EVAS [25].

Non-syndromic forms of EVA are relatively understudied [26,27]. According to Anand R et al., the morphology of the vestibular aqueduct displays a significant amount of variety [28] because relying on linear measurement of VAD will only lead to an inaccurate estimation of its size. Henceforth, volumetric measurement is necessary to assess the actual enlargement of VAD. In this retrospective study, we describe the cochlear metrics of our patients, their VAD measurements, and their inner ear/VAD volumes measured using 3D segmentation on High-Resolution Computed Tomography (HRCT). To our knowledge, no research has looked at EVA using 3D segmentation software to estimate the volume. The purpose of this study was to demonstrate a new method for the prediction of the volume of EVA by using linear measurements. In addition, we further explored the surgical outcomes of a subset of that cohort that underwent cochlear implantation surgery, specifically the development of a gusher. Finally, we validated the use of 3D segmentation in measuring VAD and the inner ear by correlating it with other VAD measurements and checking if they correlate with other cochlear metrics (cochlear dimensions, cochlear duct length).

## 2. Materials and Methods

### 2.1. Study Design

This is a single-center retrospective chart review that included MD patients managed at a tertiary cochlear implant Centre from 2009 to 2021. The patients were diagnosed by an experienced neuro-otologist using a multi-slice thin-cut high-resolution CT scan with 0.6 mm slice thickness (cochlea consisting of 1.5 turns as the middle and apical turns fuse to form a cystic apex with normal basal turn, accompanied by a dilated vestibule and enlarged vestibular aqueduct), who referred a subset of the patients to undergo cochlear implantation. The exclusion criteria were all patients with EVA with low-resolution CT scan images that could not be analyzed and those with other inner ear anomalies. The ethical approval for this study was received from the Institutional review board (IRB) (Ref. No. 22/0260/IRB). The informed consent was waived off for the study due to its retrospective nature.

### 2.2. Data Collection

**Figure 1:**
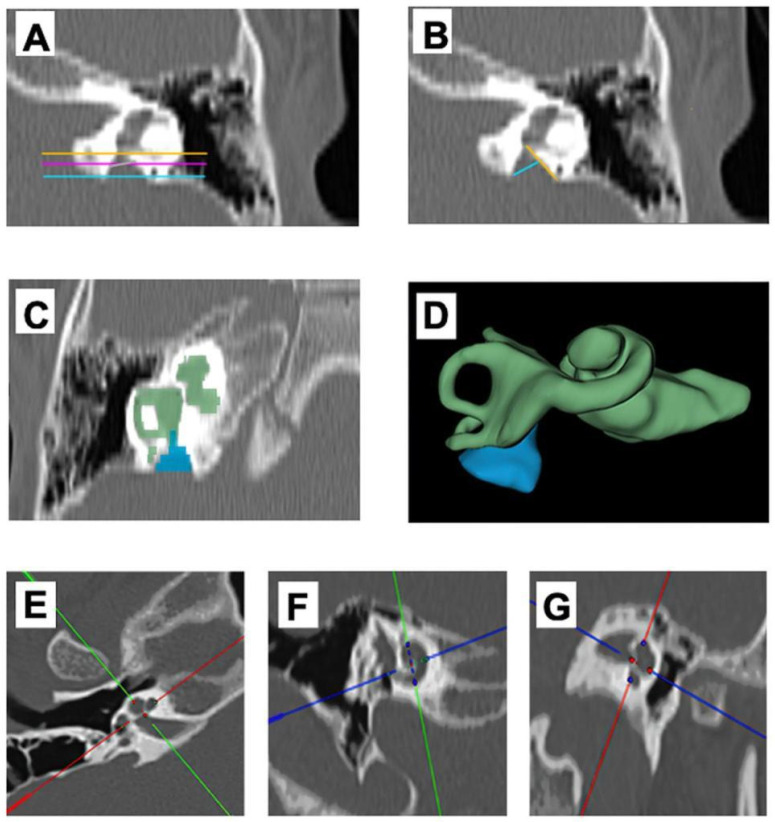
Illustration of measurements of VAD width, 3D segmentation of VAD and inner ear, and linear cochlear metrics measurements. (**A**) Left Axial CT image of temporal bone showing the method for measuring VA midpoint width, (**B**) the method for measuring VA opercular width. (**C**,**D**) showing the 3D segmentation of the inner ear and EVA. (**E**–**G**) showing cochlear metrics using OTOPLAN^®^ software.

Age was calculated using the date of performing HRCT for the non-implanted group, and for the sub-cohort undergoing surgery, age was determined at the date of surgery. In addition to age, we also collected data on the gender of each patient. Concerning imaging parameters, OTOPLAN^®^ software version 03 (developed by the collaboration of CAScination AG (Bern, Switzerland) and MED-EL (Innsbruck, Austria)) was used to measure the cochlear metrics (A value, B value, H value, Cochlear Duct Length (CDL) at the Organ of Corti (OC)) Figure 1E–G, (where A (Diameter) was the largest distance from the round window mid-point to the lateral wall of the basal turn through the modiolus; B (width) was the length of the cochlea which is a perpendicular straight line to the A-value line at the modiolus; H (height) was the distance between the center of the basal turn to the most superior apex point). VAD widths at the midpoint and at the operculum were taken following the methods of Vijayasekaran and colleagues [2] Figure 1A,B. The presence of gusher was recorded as a dichotomous variable (presence/absence) for the patients who underwent cochlear implantation.

### 2.3. Assessment and Reliability of Measurements

Two experienced neuro-otologists assessed the inner ear and VAD volume using 3D segmentation software on patients’ HRCT scans (https://www.slicer.org/, accessed on 4 April 2022) Figure 1C,D. Pearson’s correlation coefficient was used to measure the inter-rater reliability among the two readers for the inner ear volume and VAD volume.

### 2.4. Statistical Analysis

All the statistical analysis was performed using SPSS (IBM SPSS version 28.0.1.1) (Chicago, IL, USA). The categorical data were expressed in proportions. The distribution of continuous variables was checked using the Shapiro–Wilk test. Parametric variables were expressed in mean and standard deviation (SD), and non-parametric variables were expressed in median and range. The variables were further stratified by gender and the ear side. The distribution of categorical variables was compared between different groups by Chi-square tests. The distribution of parametric variables was compared between different groups using the t-test and the distribution of non-parametric variables was compared between different groups using the Mann–Whitney U test. The correlation between continuous variables was checked using Pearson’s and Spearman’s tests as per the distribution of the data. Two-way scatter plots were also used for visual checking of the correlation between continuous variables.

Stepwise multivariable linear regression was employed to check the association of the continuous outcome variable with the primary independent variable. Accordingly, the stepwise linear regression results for CT inner ear volume and CT VAD volume as the response and A value, B value, H value, CDL, VAD at the midpoint, and VAD at the operculum are used as predictors. The gender and age of the patients are used as the control variables. Moreover, at each step, in the stepwise regression, a variable included in the F statistics of the new model is significant at a 5% level of significance over the old model. Similarly, a variable is excluded from the model if the F statistics of the new model are significant at the 5% level of significance over the old model.

Confidence intervals were computed at the 95% significance level. A *p*-value below 0.05 was considered statistically significant. Odds ratios (OR) were computed for expressing effect estimates in the case of a binary outcome variable. A binary logistic regression was employed to check the association of the binary outcome variable with the main independent variable.

## 3. Results

### 3.1. Baseline Characteristics

**Figure 2:**
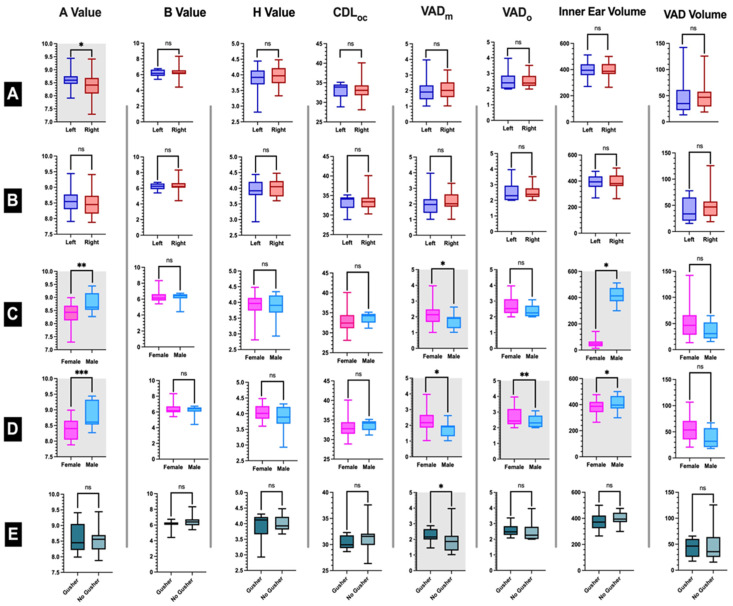
Descriptive characteristics of the study cohort. (**A**) Descriptive Statistics of the whole population (N = 42) by side of the ear (described as mean ± SD, Minimum, and Maximum, (**B**) Baseline characteristics for Cochlear Implanted ears (N = 33) by side of the ear, (**C**) Descriptive Statistics of the whole population (N = 42) by sex (described as mean ± SD, Minimum, and Maximum, (**D**) Baseline characteristics for Cochlear Implanted ears (N = 33) by sex, (**E**) Descriptive statistics of Cochlear implantation patients’ ears by Gusher outcomes, *: Significant *p*-Value (*p* ≤ 0.05), **: Significant *p*-Value (*p* ≤ 0.01), ***: Significant *p*-Value (*p* ≤ 0.001), ns: nonsignificant *p*-Value (*p* > 0.05), CDL: cochlear duct length, OC: organ of corti, VADm: vestibular aqueduct width at the midpoint, VADo: vestibular aqueduct width at the operculum). Male is colored light blue, Female is colored pink, Right is colored red, Left is colored dark blue, Gusher is colored dark green, and No gusher is colored light green.

We recruited 21 cases (42 ears), with a mean age of 13.81 (±5.10) years, including 13 females (61.9%) and 8 males (38.1%). The mean of vestibular aqueduct width in our EVA cohort was 1.9 (±0.6) mm at the midpoint and 2.53 (±0.6) mm at the operculum. The baseline characteristics of the total population are presented in Figure 2A,C. Among the 33 cochlear implanted ears with a mean age at CI of 5.15 (±5.15) years, 20 ears were for females (60.6%), whereas 13 ears were for males (39.4%). Moreover, 14 ears were left (42.4%) while 19 were right (57.6%). Figure 2B,D shows a comparison of baseline characteristics of the 33 ears by gender and side of the ear. Almost all variables were not significantly different among groups. However, only the A-value [8.36 (±0.32) versus 8.82 (±0.42)] and VAD at the midpoint [2.24 (±0.71) versus 1.69 (±0.47) mm] were significantly different between the female and male groups (*p*-Value = 0.004 and 0.008, respectively). We also found that 13 ears had a gusher (39.4%). On comparing the baseline characteristics of patients with and without gusher, we found that only VAD at the midpoint was significantly different between the two groups [2.25 (±0.42) versus 1.87 (±0.77), *p*-Value = 0.022, respectively]. Other demographics are presented in Figure 2E.

### 3.2. Assessment and Reliability of Measurements

The inter-rater reliability among the two readers for the inner ear volume (r = 0.969, *p*-Value < 0.001) and VAD volume (r = 0.841, *p*-Value < 0.001) showed both correlations are statistically significant and positive. Therefore, we verified the measurements’ reliability. However, a pairwise comparison of means showed that there is no significant difference in average CT inner ear volume (D = −0.72, *p*-Value = 0.749) between the two assessors, but there is a significant difference in average CT VAD volume (D = 8.31, *p*-value < 0.001) between the two assessors. Hence, the second assessor’s CT VAD volume measurement is consistently lower than the first. As both the assessors are consistent, we have taken the average of their measurements for CT inner ear volume and VAD volume for the rest of our study.

### 3.3. Correlations with CT VAD and Inner Ear Volumes

The results of correlation analysis show that none of the bivariate correlations of the included variables with CT inner volume were significant. On the other hand, CT VAD volume was significantly correlated with VAD at the midpoint and VAD at the operculum for combined data of both ears (r = 0.471 and 0.416, *p*-Value = 0.002 and 0.006, respectively) (Table 1). However, bivariate correlations ignore the association of the dependent variable with more than one independent variable and that may result in insignificant bivariate correlations. The predicted equations for the VAD and inner ear volumes will be as the following (where H is the cochlear height, A is the diameter of the basal turn of the cochlea, Sex is 0 for females and 1 for males, VADm is the vestibular aqueduct width at the midpoint, and VADo the vestibular aqueduct width at the operculum).

$$\mathrm{VAD}\;\mathrm{Volume}\;\left({\mathrm{mm}}^{3}\right)=-145.6+\left(2.7\times \mathrm{Age}\right)+\left(20.02\times H\right)+\left(13.61\times \mathrm{VADm}\right)+\left(19.04\times \mathrm{VADo}\right)\;\mathrm{Inner}\;\mathrm{ear}\;\mathrm{volume}\;\left({\mathrm{mm}}^{3}\right)=527.2+\left(75.2\times \;\mathrm{Sex}\right)+(5.2\;\left(5.2\times \;\mathrm{Age}\right)-\left(46.4\times A\right)+\left(35.5\times \mathrm{VADo}\right)$$

### 3.4. Regression Analysis

Table 2 shows all the results of the regression analysis presented in this section. Regarding CT inner ear volume, we found that gender, age, A-value, and VAD at the operculum were statistically significant (*p*-Value = 0.003, <0.001, 0.031, and 0.027, respectively). Regarding CT VAD volume, we found that Age, H value, VAD at the midpoint, and VAD at the operculum were significant predictors of CT VAD volume for the combined data.

### 3.5. Risk Factors for Gusher

Regression analysis of patients with and without gusher showed that only gender (OR: 0.092; 95%CI: 0.009–0.982; *p*-Value = 0.048) and VAD at the midpoint (OR: 0.106; 95%CI: 0.015–0.735; *p*-Value = 0.023) were significant predictors for the risks of gusher (Table 3).

## 4. Discussion

The present study aimed to identify the correlation between VAD volume with baseline demographics of MD patients along with their cochlear metrics and linear VAD measurements. Similarly, inner ear volume was also correlated with the same parameters. Our findings indicate that Age, H value, VAD at the midpoint, and VAD at the operculum were significant predictors of CT VAD volume. On the other hand, we found that gender, age, A-value, and VAD at the operculum can be used as significant predictors for CT inner ear volume using the data of both ears. Accordingly, we could build an algorithm to help us predict both CT inner ear volume and VAD volumes in these patients.

For instance, the significant positive regression coefficient of gender shows that the CT inner ear volume is higher for males than females when adjusted for other variables. The CT inner ear volume also increases significantly by 5.2 for a one-year increase in the patient’s age. CT inner ear volume decreases by 46.4 mm^3^ for a 1 mm increase in A value. On the other hand, CT inner ear volume increases by 35.5 mm^3^ for a 1 mm increase in VAD at the operculum.

We could also establish an equation for estimating CT VAD volumes in our patients based on the regression analysis findings. In this context, CT VAD volume increases significantly by 2.7 for a one-year increase in the age of the patient. According to Anand R et al. [28], EVA volumetric measures were strongly correlated to the linear measurements of both midpoint length and operculum size. We found the same results as CT VAD volume increases significantly by 13.61 and 19.04 for 1 mm increases in VAD at the midpoint and VAD at the operculum, respectively, as keeping both of them increases the predictability.

Following the first presentation of MD by Carlo Mondini in the 1970s, which was conducted through anatomic dissection, many investigations followed to describe and study the disease in more detail worldwide [13,14]. Here, we enrolled 21 patients (42 ears) with MD and performed cochlear implantation on a subset of 33 ears. In addition, we retrospectively checked the patients’ data for ear side-associated differences, gender-associated differences, and differences associated with the development of gusher. Even though there were no significant differences among the ages of males and females in our cohort, males consistently showed significantly higher A values compared to their female counterparts. In contrast, females had significantly larger VAD volumes and higher VAD widths at the midpoint. Ruthberg J et al. found the same in their US cohort; there were larger VA metrics in females than males [29], indicating that this pattern could be generalized. The difference in the A value between the right and left ears did not carry on to the surgery sub-cohort, suggesting that the achieved significance may have been due to chance.

MD patients are at particularly high risk for developing gusher [22,30], and recurrent meningitis [31,32]. Papsin et al. reported that gushers could present in up to 6.7% of cases with inner ear malformations [33]. In contrast, in their more recent work, Hashemi and colleagues reported a lower percentage of 5.58% [34]. Compared with these findings, we found that the rate of gusher in our included CI population was 39.4%, which is remarkably higher than the previously reported rates but it is worth noting that all of the study cases are at high risk for gusher. Therefore, it is necessary to understand and predict the development of gusher and manage it properly. Our findings indicate that gender and VAD width at the midpoint can be significant predictors of the risks of gusher outcomes. While that suggests that gender and VAD width at the midpoint can help surgeons predict the risk of gusher before operating, larger multicenter studies are needed to validate that claim. Hashemi and colleagues [34] found that gusher was associated with having more severe structural abnormalities, whereas Dettman et al. [30] found that gusher significantly correlates with partial electrode insertion. We believe that our work is the first to show the association of gusher with VAD length at the midpoint. Therefore, we recommend that this association is checked in future work to establish reliability. We hypothesized that there was a correlation between VAD and inner ear metrics in support of the mechanical pressure theory stated by Jackler and colleagues [14] and supported by others [35,36]. Our data did not support that hypothesis, which further undermines this theory in favor of the genetic theory adopted by Mohnish and others [37,38,39]. We recommend that patients’ genetic backgrounds be studied in future works to improve patient care and enable earlier diagnosis.

Our study has some limitations to be considered before interpreting the current findings. First, the sample size is small. Second, the data were collected retrospectively. These might interrupt the integrity of the current data and cause selection bias in included participants. This might also limit the ability to predict different risk factors, which will need a better allocation of patients in future studies. Moreover, some demographics were statistically significant among the different groups in this study, which might also represent a degree of bias when analyzing the factors predicting the reported outcomes. Therefore, future relevant investigations overcoming these limitations are needed.

## 5. Conclusions

Our findings indicate that age, H value, VAD at the midpoint, and VAD at the operculum were significant predictors for CT VAD volume. On the other hand, we found that sex, age, A-value, and VAD at the operculum can be used as significant predictors for CT inner ear volume using data from both ears. In addition, patients’ gusher outcomes were significantly differentiated by sex and VAD length at the midpoint. While our data did not support the mechanical pressure theory stated by Jackler and colleagues, we recommend that future studies are carried out to provide more insight into the etiology of MD in our population.

## Figures and Tables

**Table 1:** Correlation of different variables with CT inner and VAD volumes.

Variables	CT Inner Volume		CT VAD Volume	
	Combined		Combined	
	R	*p*-Value	R	*p*-Value
Age	0.292	0.061	0.278	0.075
A Value	−0.044	0.783	0.015	0.926
B Value	0.183	0.246	0.048	0.765
H Value	−0.154	0.33	0.231	0.141
CDL at the level of OC	0.092	0.563	0.053	0.74
VAD at midpoint (mm)	−0.158	0.318	0.471	**0.002**
VAD at operculum (mm)	0.019	0.903	0.416	**0.006**

Significant *p* values are shown in bold. Constant: Y-intercept, CDL: cochlear duct length, OC: organ of corti, VAD: Vestibular aqueduct.

**Table 2:** Regression analysis of factors predicting CT inner volume and CT VAD volume.

Variable	Coefficient	Std Error	t-Value	*p*-Value
CT inner volume for combined data				
Constant	527.203	163.229	3.23	0.003 *
Sex	75.202	19.085	3.94	<0.001 *
Age	5.188	1.605	3.232	0.003 *
A Value	−46.35	20.618	−2.248	0.031 *
VAD at Operculum (mm)	35.474	15.356	2.31	0.027 *
CT VAD volume for combined data				
Constant	−145.593	44.320	−3.285	0.002 *
Age	2.744	0.683	4.016	<0.001 *
H Value (Height)	20.017	9.595	2.086	0.044 *
VAD at Midpoint (mm)	13.609	6.296	2.161	0.037 *
VAD at Operculum (mm)	19.039	7.364	2.585	0.014 *

Significant *p* values are shown in bold. CDL: Constant: Y-intercept, CDL: cochlear duct length, OC: organ of corti, VAD: Vestibular aqueduct, *: Statistically significant *p*-Value (*p* ≤ 0.05).

**Table 3:** Logistic regression analysis to model the chance of no gusher.

Variable	Coefficient	OR	*p*-Value	95%C.I.	
				Lower	Upper
Constant	−13.608	0	0.152		
Sex	−2.384	0.092	0.048 *	0.009	0.982
CDL at the level of OC	0.64	1.897	0.074	0.939	3.833
VAD at midpoint (mm)	−2.242	0.106	0.023 *	0.015	0.735

Significant *p* values are shown in bold. CDL: cochlear duct length, OC: organ of corti, VAD: Vestibular aqueduct, *: Statistically significant *p*-Value (*p* ≤ 0.05).

## Data Availability

The data presented in this study are available on request from the corresponding author. The data are not publicly available due to the ethical approval agreement.
